# Myostatin and Insulin-Like Growth Factor I: Potential Therapeutic Biomarkers for Pompe Disease

**DOI:** 10.1371/journal.pone.0071900

**Published:** 2013-08-14

**Authors:** Yin-Hsiu Chien, Der-Sheng Han, Wuh-Liang Hwu, Beth L. Thurberg, Wei-Shiung Yang

**Affiliations:** 1 Department of Medical Genetics, National Taiwan University Hospital, Taipei, Taiwan; 2 Department of Physical Medicine and Rehabilitation, National Taiwan University Hospital, Taipei, Taiwan; 3 Department of Internal Medicine, National Taiwan University Hospital, Taipei, Taiwan; 4 Department of Physical Medicine and Rehabilitation, National Taiwan University Hospital, BeiHu Branch, Taipei, Taiwan; 5 Graduate Institute of Clinical Medicine, College of Medicine, National Taiwan University, Taipei, Taiwan; 6 Research Center for Developmental Biology and Regenerative Medicine, National Taiwan University, Taipei, Taiwan; 7 Department of Pathology, Genzyme, a Sanofi company, Framingham, Massachusetts, United States of America; Universidad Europea de Madrid, Spain

## Abstract

**Objective:**

Myostatin and insulin-like growth factor 1 (IGF-1) are serum markers for muscle growth and regeneration. However, their value in the clinical monitoring of Pompe disease – a muscle glycogen storage disease – is not known. In order to evaluate their possible utility for disease monitoring, we assessed the levels of these serum markers in Pompe disease patients receiving enzyme replacement therapy (ERT).

**Design:**

A case-control study that included 10 patients with Pompe disease and 10 gender- and age-matched non-Pompe disease control subjects was performed in a referral medical center. Average follow-up duration after ERT for Pompe disease patients was 11.7 months (range: 6–23 months). Measurements of serum myostatin, IGF-1, and creatine kinase levels were obtained, and examinations of muscle pathology were undertaken before and after ERT in the patient group.

**Results:**

Compared with control subjects, Pompe disease patients prior to undergoing ERT had significantly lower serum IGF-1 levels (98.6 ng/ml vs. 307.9 ng/ml, *p* = 0.010) and lower myostatin levels that bordered on significance (1.38 ng/ml vs. 3.32 ng/ml, *p* = 0.075). After ERT, respective myostatin and IGF-1 levels in Pompe disease patients increased significantly by 129% (from 1.38 ng/ml to 3.16 ng/ml, *p* = 0.047) and 74% (from 98.6 ng/ml to 171.1 ng/ml, *p* = 0.013); these values fall within age-matched normal ranges. In contrast, myostatin and IGF-1 serum markers did not increase in age-matched controls. Follistatin, a control marker unrelated to muscle, increased in both Pompe disease patients and control subjects. At the same time, the percentage of muscle fibers containing intracytoplasmic vacuoles decreased from 80.0±26.4% to 31.6±45.3%.

**Conclusion:**

The increase in myostatin and IGF-1 levels in Pompe disease patients may reflect muscle regeneration after ERT. The role of these molecules as potential therapeutic biomarkers in Pompe disease and other neuromuscular diseases warrants further study.

## Introduction

Pompe disease is a lysosomal storage disorder in which a deficiency of acid α-glucosidase causes glycogen accumulation in all tissues, particularly cardiac and skeletal muscle. Microscopic analyses of muscle tissue typically show accumulation of glycogen-containing vacuoles in the myocytes [1]. Pompe disease presents with a wide spectrum of phenotypes, ranging from a severe and rapidly progressive form with infantile-onset (IOPD) to a form that is slowly progressive with late-onset (LOPD). Enzyme replacement therapy (ERT) with recombinant human alglucosidase alfa (Myozyme^®^, Genzyme, Cambridge, MA) prolongs survival and reverses cardiomegaly in IOPD [2], [3]. Patients with IOPD diagnosed through newborn screening have the best outcomes for ERT [4]. In LOPD, although ERT has been associated with positive responses in motor capability and pulmonary function, there is significant variability in outcomes [5].

Due to advances leading to prolonged life expectancy in children with Pompe disease, rehabilitation services are needed to maximize functional status, prevent airway obstruction, facilitate patients’ ability to communicate, and improve respiratory function [6]. In overcoming variation in patient outcomes with ERT in Pompe disease, the identification of non-invasive biomarkers would be a step forward for effective monitoring of clinical progress. Serum creatine kinase (CK) is a non-specific marker for muscle damage, but it has not been shown to be useful for monitoring treatment effects. A glucose tetrasaccharide, Glcα1-6Glcα1-4Glcα1-4Glc, is elevated in the urine of IOPD patients and can be used as a monitoring marker [7], although it has not been thoroughly validated.

Insulin-like growth factor 1 (IGF-1) – which is a downstream target of growth hormone – increases protein synthesis in skeletal muscle through the Akt-mTOR pathway. Indeed, transgenic mice over-expressing IGF-1 have accelerated skeletal muscle regeneration [8]. Moreover, IGF-1 expression was elevated in a rat model of cardiomyocyte hypertrophy and in healthy volunteers following strength training [9], [10]. On the other hand, myostatin (a potent myokine that belongs to the transforming growth factor-β superfamily) prevents skeletal muscle over-growth. Skeletal muscle mass was greater in myostatin-deficient mice compared with wild-type animals [11]. Interestingly, nutrient supply elevates mRNA expression levels in chicken skeletal muscle for both skeletal muscle regulators, IGF-1 and myostatin [12].

We previously proposed a “yin and yang” (or accelerator-brake) mechanism for myostatin and IGF-1 in the regulation of muscle mass or function [13]. Based on this accelerator-brake model, we hypothesized that with ERT (which removes glycogen and regenerates muscles), both IGF-1 and myostatin levels would increase. We have therefore conducted a case-control study to monitor these serum markers in Pompe disease patients receiving ERT. We demonstrate a significant increase in IGF-1 and myostatin levels after ERT.

## Methods

### Human Subjects

Ten consecutive patients with Pompe disease, diagnosed at the National Taiwan University Hospital, were recruited for this study. Written informed parental consent was obtained for all patients prior to enrollment. Blood samples and muscle biopsies were taken before and after ERT initiation. Blood samples taken before or within four weeks of ERT initiation were considered pre-ERT. Average ERT duration was 11.7 months (range: 6–23 months). Our study population contained six IOPD patients and four LOPD patients. Among the six IOPD patients, four were identified through a newborn screening program and received ERT before they reached one month of age [4], while two were identified clinically and started ERT at the ages of two and four months. The two clinically-identified patients underwent tracheostomy with subsequent 24-hour ventilatory support; one of these patients later died at the age of four years. The four patients identified through newborn screening have remained ventilator-independent for at least four years after ERT initiation. The four LOPD patients presented with abnormal liver function tests or muscle weakness, and the age range for starting ERT among these patients was between 10 and 25 years. None of these patients required ventilator support at the time of this study and all of them could walk independently.

All Pompe disease patients received ERT with recombinant human alglucosidase alfa (Myozyme^®^, Genzyme, Cambridge, MA), administered intravenously at a dose of 20 mg/kg every 14 days, following diagnosis of Pompe disease. Ten age- and gender-matched subjects, who received blood tests for reasons not related to muscle diseases, were recruited and served as controls. This control group included patients with hyperphenylalaninemia, galactosemia, and newborns with abnormal results following newborn screening for inborn errors of metabolism such as citrullinemia or argininemia. The major reason for having a control group was to demonstrate age-associated changes in myostatin and IGF-1 levels in non-Pompe disease children. The control subjects received neither ERT nor underwent muscle biopsy. This study was approved by the Institutional Review Board of the National Taiwan University Hospital.

### Biochemistry and Histology Analysis

Serum myostatin was measured with an ELISA kit (Immunodiagnostik, Bensheim, Germany) with a minimum detection limit of 270 pg/ml, and respective intra- and inter-assay variability of less than 10% and 15% [14]. The test employed a polyclonal antibody against full-length myostatin peptide with a competitive immunoassay technique. In the pre-treatment process, serum samples were thawed and diluted two-fold with dilution buffer (provided in the ELISA kit). Serum IGF-1 was measured with the Mediagnost ELISA kit (Reutlingen, Germany). Minimum detection limit, and intra- and inter-assay variability for the IGF-1 ELISA kit were 90 pg/ml, 6.7%, and 6.8%, respectively [15]. This kit is not only highly specific and sensitive, but also has a small sample volume requirement, making it ideal for pediatric patients. Serum follistatin was analyzed with a sandwich ELISA kit (Quantikine^®^, R&D Systems, Minneapolis, MN, USA). Minimum detection limit, and intra- and inter-assay variability for this kit were 29 pg/ml, 2.4%, and 7.1%, respectively [16]. The absorption of each well was read using a VersaMAX tunable microplate reader (Molecular Devices, Sunnyvale, CA, USA) at 450 nm against 620 nm as a reference. The four-parameter logistic regression model was employed to calculate the concentrations with optical density values [17]. Creatine kinase was measured in a hospital laboratory with an automated analyzer (AU-5800, Beckman Coulter, Tokyo, Japan).

The Peabody Developmental Motor Scale, Second Edition (PDMS-II) was employed by a physical therapist to evaluate the gross and fine motor development of infantile patients [4].

For treatment monitoring, incisional quadriceps muscle biopsies were taken before and six months after ERT from both legs of each IOPD patient. Each tissue specimen was cut in half and the separate halves were subjected to different processing methods. One half was fixed in 10% neutral buffered formalin, processed into paraffin blocks, sectioned and stained with hematoxylin and eosin. In these sections, the glycogen load, indicated by the percentage of muscle fibers containing intracytoplasmic vacuoles under high power field, was calculated by an experienced pathologist using the formula: glycogen load = (number of muscle fibers containing intracytoplasmic vacuoles×100%)/number of total muscle fibers. The other half of the specimen was fixed in 3% glutaraldehyde, processed into epoxy resin blocks, sectioned into one-micron sections and stained with a combination of periodic acid-Schiff stain and Richardson’s stain for high resolution light microscopy (HRLM), as previously described [18]. Glycogen load in these sections was measured by computer morphometry with MetaMorph software and expressed as the percentage of tissue area occupied by glycogen, as previously described [18].

### Statistical Analysis

The test of means between Pompe disease and non-Pompe disease groups was performed with the Wilcoxon rank-sum test, and the effect of ERT on serum markers was tested with the Wilcoxon signed-rank test. All significance levels were set at *p*<0.05. Statistical analyses were performed with SPSS^®^ 11.5 software (SPSS Inc. Chicago, Illinois, USA).

## Results

Ten Pompe disease patients and 10 control subjects were included in this study. There was no difference in age or gender ratio between patient and control groups. At study baseline, Pompe disease patients had significantly lower serum IGF-1 levels than control subjects (98.6 ng/ml vs. 307.9 ng/ml, respectively, *p* = 0.010), in addition to lower myostatin levels with borderline significance (1.38 ng/ml in Pompe disease vs. 3.32 ng/ml in control subjects, *p* = 0.075). Serum follistatin levels (which served as an internal control) were also lower in Pompe disease patients, but were not significantly different from matched control subjects (Table 1).

**Table 1:** Demographic data and baseline biomarker levels for control subjects and Pompe disease patients. Data are expressed as mean (SD).

	Control	Pompe	*p* value
**N**	10	10	
**Gender (male/female)**	6/4	6/4	1.000
**Age at initial visit (range in months)**	78.0 (1–300)	76.5 (1–300)	0.267
**Myostatin (ng/ml)**	3.32 (3.31)	1.38 (1.29)	0.075
**IGF-1 (ng/ml)**	307.9 (142.5)	98.6 (108.8)	0.010
**Follistatin (ng/ml)**	1.18 (0.48)	0.87 (0.29)	0.101
**Muscle fiber with vacuoles (%)**	–	80.0 (26.4)	
**Creatine kinase (U/L)**	–	972 (591)	

**Figure 1:**
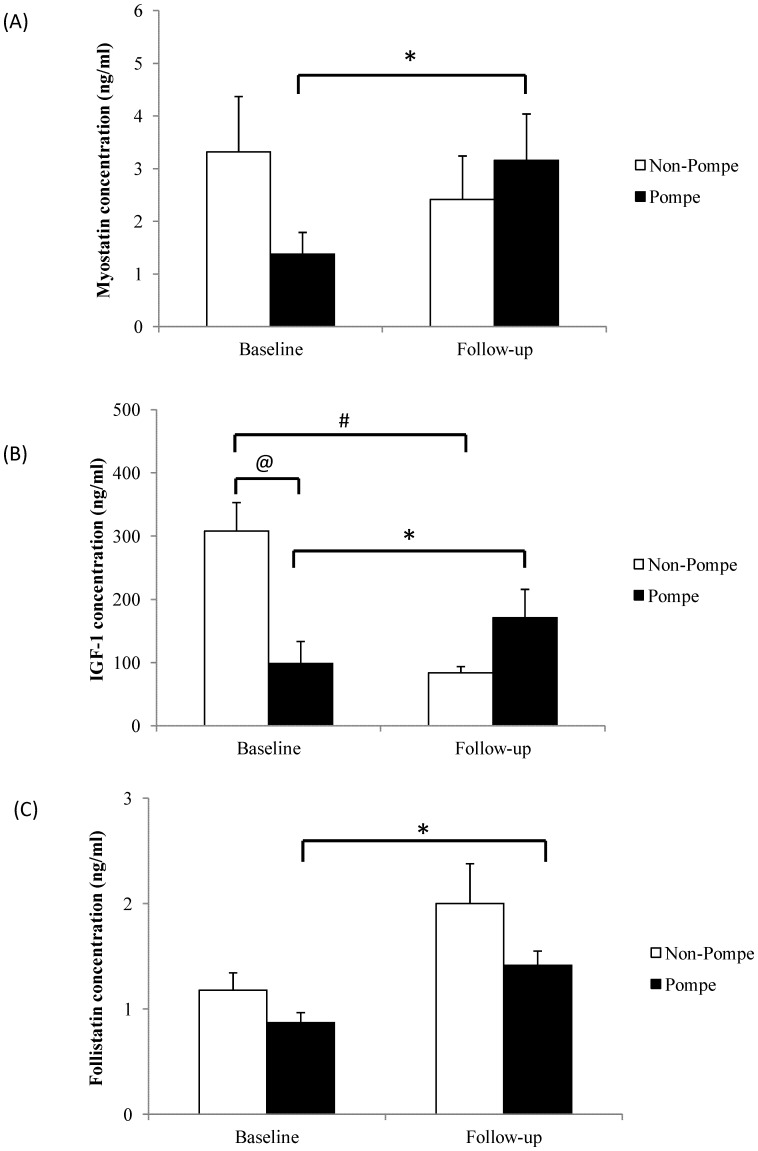
Serum myostatin (A) and IGF-1 (B) levels for control subjects and Pompe disease patients. The error bars represent the standard error of means. @ denotes *p*<0.05 between control subjects and Pompe disease patients at baseline. # and * denote *p*<0.05 between baseline and follow-up for respective control subjects and Pompe disease patients. The control group did not receive enzyme replacement therapy.

**Figure 2:**
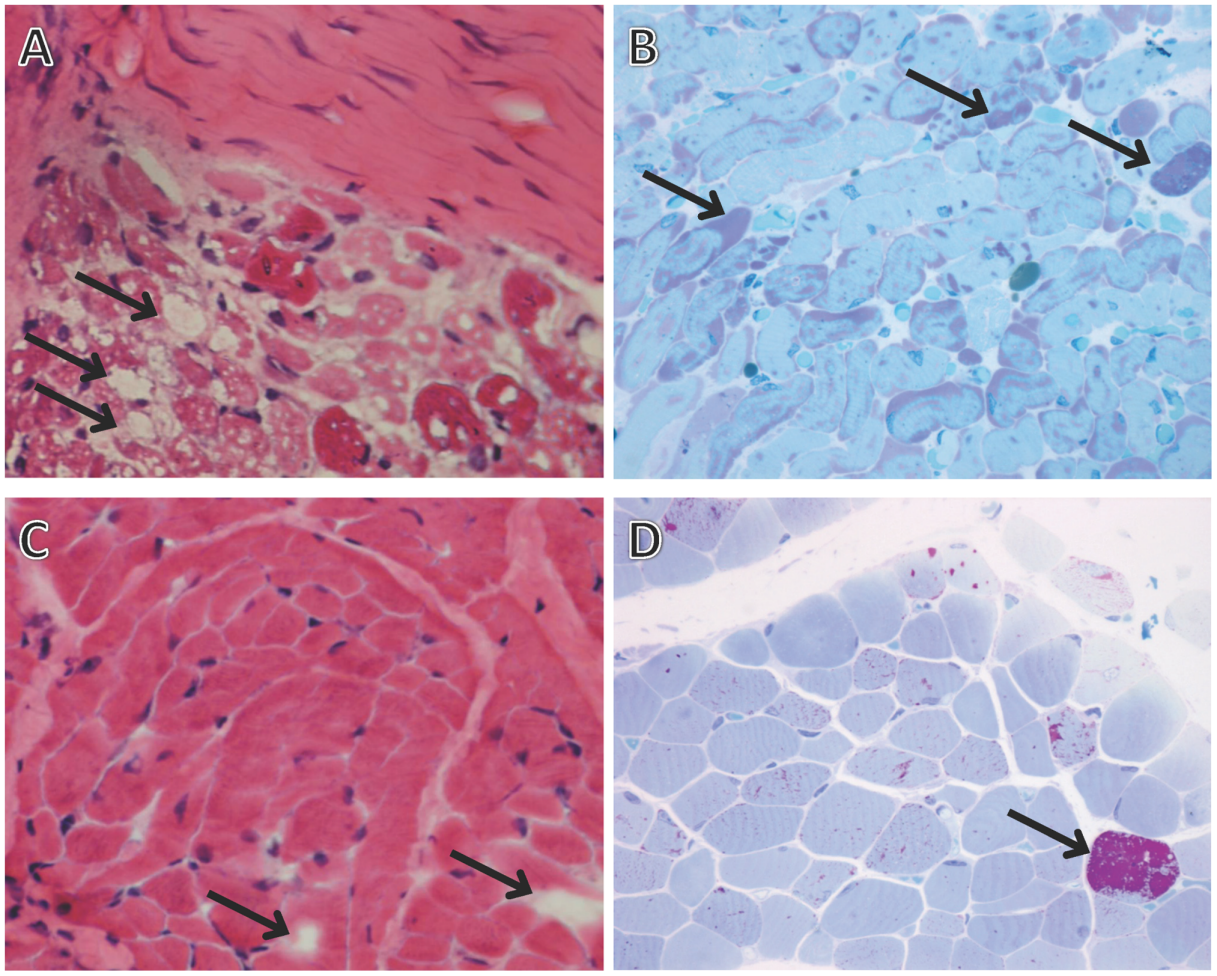
Representative microscopic images of muscle pathology for a patient with infantile-onset Pompe disease. (A) Tissue samples, obtained before ERT at age 10 days, processed in paraffin and stained with hematoxylin and eosin: numerous large empty intracytoplasmic vacuoles (indicated by arrows) are present in the muscle fibers. (B) Muscle tissue corresponding to same origin as in (A) (obtained before ERT at age 10 days) but processed in epoxy resin for high-resolution light microscopy (HRLM) and stained with periodic acid-Schiff/Richardson’s stain. Note the deep purple glycogen granules (arrow). (C) Few empty vacuoles (arrows) were observed in the muscle fibers (processed in paraffin and stained with hematoxylin and eosin) from the same patient after ERT for 6 months. (D) Muscle tissue obtained post-ERT corresponding to the same origin but processed in epoxy resin for HRLM and stained with periodic acid-Schiff/Richardson’s stain. Few purple glycogen granules (arrow) remained after ERT treatment.

We then compared serum myostatin and IGF-1 levels before and after ERT in Pompe disease patients; both these markers increased significantly after ERT for 12 months. Levels of serum myostatin in Pompe disease patients increased significantly by 129% (from 1.38 ng/ml to 3.16 ng/ml, *p* = 0.047). In contrast, myostatin levels at follow-up for control subjects (i.e. those who did not receive ERT) were not significantly different from baseline (2.42 ng/ml at follow-up vs. 3.32 ng/ml at baseline; Figure 1A). For IGF-1, levels increased by 73.5% in Pompe disease patients after ERT (from 98 ng/ml at baseline to 171 ng/ml at follow-up, *p* = 0.013), whereas in age-matched control subjects, the levels declined (from 307 ng/ml at baseline to 83 ng/ml at follow-up, *p* = 0.027; Figure 1B). The follistatin level increased by 62.2% in Pompe disease patients, and also increased (but not to a significant extent) in control subjects (Figure 1C). In the six IOPD patients, the percentage of muscle fibers containing intracytoplasmic vacuoles in paraffin hematoxylin-eosin sections decreased from 80.0±26.4% to 31.6±45.3% after ERT (Figures 2A and 2C). Some regenerative myocytes were occasionally seen in muscle specimens after ERT. The pre- and post-ERT levels for CK were not significantly different (972±591 U/L vs. 893±506 U/L, *p* = 0.721). However, although some patients showed improvement in motor development milestones, the PDMS-II quotient after ERT still showed a significant decline (from 95.2±8.1 to 79.5±18.6, *p* = 0.046).

## Discussion

In patients with Pompe disease, we demonstrated lower baseline levels of serum IGF-1 compared with control subjects and elevations in serum IGF-1 and myostatin levels following ERT. The significant increases in serum levels of myostatin and IGF-1 might be related to muscle regeneration, and could also correspond with muscle recovery as shown in pathological examinations.

IGF-1 is an age-related serum protein that has insulin-like metabolic activities and growth-controlling properties [19]. IGF-1 expression counteracts muscle function decline in mouse models of muscular dystrophy [20] and amyotrophic lateral sclerosis [21], activates satellite cells, and improves the survival of motor neurons. Higher IGF-1 levels at baseline in control subjects might reflect the stage of life that is consistent with rapid muscle growth, while subsequent decreases at follow-up could be caused by slow-down in muscle growth with older age. In the present study, the baseline IGF-1 level of Pompe disease patients was lower than for control subjects. Lack of regeneration signals that activate satellite cells – due to muscular destruction caused by glycogen accumulation – could be one explanation for this finding. After completion of ERT, we found that glycogen accumulation had decreased, and that IGF-1 expression had increased to approach normal levels.

IGF-I levels are highly age-dependent in children. Immediately following birth, infants have a prominent postnatal surge in circulating IGF-1 levels [22]. The level then declines to reach a nadir before one year of age, but increases slowly thereafter, surging again in adolescence [23], [24]. In our study, the serum level of IGF-1 in the control group declined during follow-up. This is to be expected as most of the IOPD patients in our study started ERT before one month of age, representing a time-window when IGF-1 levels in normal controls would be at their highest. In addition, the four LOPD patients who started ERT during adolescence had higher reference IGF-1 values. By including age-matched controls in our study, we were able to minimize sample heterogeneity caused by age-associated differences in marker levels.

Previously, we proposed an “accelerator-brake model” to illustrate the role of myostatin in regulating muscle growth and function [13]. Myostatin serves as a negative feedback molecule during the processes of muscular growth and regeneration in order to limit the final muscle mass [9], [25]. In this model, myostatin is switched on only during muscle growth or regeneration. In the Pompe disease patients included in this study, baseline myostatin levels were lower than in control subjects at a level that approached statistical significance (*p* = 0.075), suggesting a low level of muscle growth or regeneration. After ERT completion, myostatin levels in the Pompe disease patients increased significantly. This elevation in myostatin may have occurred as a signals of muscle regeneration [13]. ERT stops the process of muscular destruction in order that new muscle fibers can be generated [26]. Since myostatin negatively regulates human myoblast proliferation, antagonism of myostatin may enhance the therapeutic effect of ERT in those patients who respond poorly [27].

Follistatin was originally found in the ovarian follicular fluid, and broader expression was later shown in the reproductive, endocrine, digestive, and neurological systems of humans [28], [29]. Follistatin binds to members of the transforming growth factor-β superfamily, and exerts an inhibitory effect on these growth factors [30]. The follistatin reference value for a term neonate is 0.43±0.02 ng/ml, which is correlated with fat mass and gestational age [31]. In our study, baseline serum levels of follistatin were higher than normal for both the Pompe disease group and the control group, and increased at follow-up. A possible explanation could be high inter-individual variation resulting from a small sample size. In addition, because follistatin augments adipogenesis, higher adipose tissue growth might correlate with higher follistatin serum levels [29]. Follistatin is a cytokine that does not originate in muscle; we therefore regarded it as a control marker.

This study has some limitations. Due to the rarity of the disease, the sample was small and heterogeneous and duration of follow-up was short. However, we have still obtained positive results from a small sample size. By recruiting more patients for a larger sample size, it should be possible to obtain more detailed information in the future through subgroup analyses, e.g. by looking at IOPD versus LOPD or good responders versus poor responders. In addition, due to ethical considerations, the present study does not include a Pompe disease cohort that did not receive ERT (which would have been the ideal choice for the control group). Therefore, it is not known if the effects are due to the treatment or related to the disease’s natural course. Furthermore, assessments of muscle mass and strength may be needed to delineate the correlation between muscle regeneration and serum marker levels. A future longitudinal study is also needed to analyze dynamic changes in these markers over the disease course.

In conclusion, serum IGF-1 levels were significantly lower in Pompe disease patients relative to normal control subjects, and serum myostatin also showed a trend for lower levels. Moreover, expression levels for both these markers reached normal values after ERT in Pompe disease patients. In addition to shedding light on the pathogenesis of neuromuscular disease, these molecules represent potential new therapeutic markers.
